# Seasonal changes of prokaryotic microbial community structure in Zhangjiayan Reservoir and its response to environmental factors

**DOI:** 10.1038/s41598-024-55702-5

**Published:** 2024-03-06

**Authors:** Xintao Yu, Yong Li, Yue Wu, Hui Gao, Wei Liu, Huan Liu, Sidan Gong, Honglian Wu

**Affiliations:** 1 Faculty of Geosciences and Environmental Engineering, Southwest Jiaotong University, Chengdu, 610059 China; 2 Sichuan Aqua Gathering Eco-environment Management Co., Ltd., Neijiang, 641000 China; 3 Eastern Newly Developed Area Water Conservany Administrative Station, Pihe River Administrative Office, Sichuan Du Jiangyan Water Conservancy Development Center, Chengdu, 641400 China

**Keywords:** Environmental impact, Limnology

## Abstract

As a typical sub-deep reservoir in the upper reaches of the Yangtze River in the southwest region, Zhangjiayan Reservoir is also an important source of drinking water. Exploring the role of microorganisms in the material cycle of water bodies is of great significance for preventing the exacerbation of eutrophication in the reservoir. In this study, water samples from the overlying water of five points in the reservoir were collected four times in spring (April), summer (July), autumn (November), and winter (January) of 2022–2023 using a gas-tight water sampler. Physicochemical factors were measured, and the microbial community structure was analyzed by high-throughput MiSeq sequencing of the V3–V4 hypervariable region of 16S rRNA gene in order to explore the relationship between physicochemical factors and microbial community structure and the dominant microbial populations that affect eutrophication of the reservoir. The following results were obtained through analysis. Among the 20 overlying water samples from Zhangjiayan Reservoir, a total of 66 phyla, 202 classes, 499 orders, 835 families, 1716 genera, and 27,904 ASVs of the bacterial domain were detected. The phyla *Proteobacteria* and *Actinobacteria* were dominant in the microbial community of the overlying water in Zhangjiayan Reservoir. At the genus level, *hgcI_clade* and *Actinobacteria* had the highest abundance and was the dominant population. The microbial community in the water of Zhangjiayan Reservoir has a high level of diversity. The diversity index ranked by numerical order was winter > autumn > summer > spring. Significant differences were found in the composition and structure of the microbial community between the spring/summer and autumn/winter seasons (*p* < 0.05). Total phosphorus, dissolved total phosphorus, soluble reactive phosphorus, and dissolved oxygen have a significant impact on the composition and structure of the microbial community (*p* < 0.01). The bacterial community in the overlying water of Zhangjiayan Reservoir showed a mainly positive correlation. *Sphingomonas*, *Brevundimonas*, and *Blastomonas* were the central populations of the bacterial community in the overlying water of Zhangjiayan Reservoir. This study indicates that environmental factors, such as phosphorus and other nutrients, have a significant impact on the formation of the microbial community structure in different seasons. *Sphingomonas*, *Brevundimonas*, and *Blastomonas* are key populations that may have a significant impact on eutrophication in Zhangjiayan Reservoir.

## Introduction

Microorganisms in the overlying water of lakes are an important component of lake ecosystems. The diversity and composition of these microorganisms play a crucial role in the material cycling and energy flow processes at the sediment–water interface. They primarily participate in the decomposition of organic matter and mineralize it into inorganic compounds that can be utilized by plants for primary production^1^. The structure and diversity of microbial communities are closely related to the nutrient cycling in aquatic ecosystems. Therefore, deciphering the coupled relationship between factors causing eutrophication and the microbial community in the surface waters of lakes and reservoirs is of great significance for controlling eutrophication.

Currently, the study of the correlation of microorganisms in eutrophic lakes has become a hot topic among scholars. Shen et al.^2^ examined the relationship between the nutritional status of freshwater lakes in the Yungui Plateau in China and the community of planktonic microorganisms using metagenomic analysis. Zhang et al.^3^ analyzed the diversity and distribution of bacterial communities in the contaminated saltwater lake Bositeng Lake in Xinjiang, as well as the response of microorganisms to environmental changes in stable inland water bodies in these arid or semi-arid regions. Liu et al.^4^ investigated the microbial communities in surface water of nine high altitude lakes on the Yunnan Plateau during both the dry and rainy seasons. It was found that the structure of the microbial communities in eutrophic water bodies was related to the nutrient level.

Although there have been some studies focusing on the microbial community diversity, composition, and variation in lakes, most of the research has been conducted on deep plateau lakes (Qinghai Lake^5^ and Fuxian Lake^6^), shallow water lakes (Taihu Lake^7^ and Poyang Lake^8^), northern lakes (Beihai Lake^9^ and Miyun Reservoir^10^), and southern lakes (Dongting Lake^11^ and Chaohu Lake^12^). Currently, there is limited research on the microbial community diversity and structure of the subdeep lakes in the southwestern region. Li et al.^13^ have already conducted research on the microbial community structure in the sediment of sub-deep lake (Sancha Lake) in the southwest region, and its relationship with eutrophication environmental factors. However, studies on the microbial community structure in the lake water, particularly in the overlying water, and its association with environmental factors are scarce.

The Zhangjiayan Reservoir (104°17′16″–104°18′45″E, 30°25′33″–30°26′12″N) is located within the boundaries of Gaoming Town in the eastern new district of Sichuan Province, China. It is situated within the Zhangjiayan River, a tributary of the Jiangxi River in the Tuo River system, and is classified as a typical sub-deep reservoir in the southwestern region. Zhangjiayan Reservoir has a surface area of 86.24 m^2^ with an average depth of 16.5 m. The shore of the reservoir is winding and its shape is quite complex. The region where Zhangjiayan Reservoir is located belongs to the humid subtropical monsoon climate of Central Asia, with rainfall mainly concentrated in July to August. The reservoir water source includes natural rainfall as well as the main stream of the Minjiang River from the western Sichuan Plateau. The period from March to July every year is the period of agricultural irrigation and drainage. Due to the impact of the rainy season and agricultural irrigation and drainage, the flow rate of water entering and leaving the reservoir area is relatively fast during the spring and summer seasons. There is no point source pollution from residential or industrial areas within the catchment area, and there is relatively less non-point source pollution. The Zhangjiayan Reservoir is an essential source of drinking water and provides life-sustaining water to over 300,000 people in the downstream city of Jianyang. In recent years, the water quality of Zhangjiayan Reservoir has met the Surface water Class III water standard except for total phosphorus, but the water body has become eutrophic and seasonal blue-green algae phenomena have occurred. For many years, phosphorus has been the limiting factor for eutrophication in Zhangjiayan Reservoir^14^. Currently, Zhangjiayan Reservoir is experiencing mild eutrophication, which may further exacerbate the risk of eutrophication. Therefore, further exploration of the role of microorganisms in the material cycling process of the reservoir, especially in the phosphorus cycle, is of great significance for the restoration and management of eutrophication in Zhangjiayan Reservoir.

Based on the above research background, we propose the following hypotheses: the diversity of microorganisms in the overlying water of Zhangjiayan is abundant. The microbial structure composition of the overlying water of Zhangjiayan undergoes significant seasonal changes in response to environmental factors. There are key species in the overlying water of Zhangjiayan, which play an important role in community stability and interspecies interactions, especially in material cycling processes, particularly in phosphorus cycling.

In light of the above research objectives, this study employed environmental physicochemical factor measurement, DNA extraction, PCR amplification, and high-throughput sequencing to analyze the diversity, structure, temporal and spatial distribution of bacterial communities in the overlying water of Zhangjiayan Reservoir and their relationship with eutrophication factors. We attempt to investigate the major environmental factors that influence the structure of microbial communities, and uncover the microbial populations that have a significant impact on eutrophication in Zhangjiayan Reservoir. This has important research significance for the restoration of eutrophic water bodies in Zhangjiayan Reservoir and the protection of water resources. Its research results can provide a scientific theoretical basis for the restoration and management of eutrophication in similar lakes and reservoirs in China.

## Materials and methods

### Research area and sample collection

**Figure 1:**
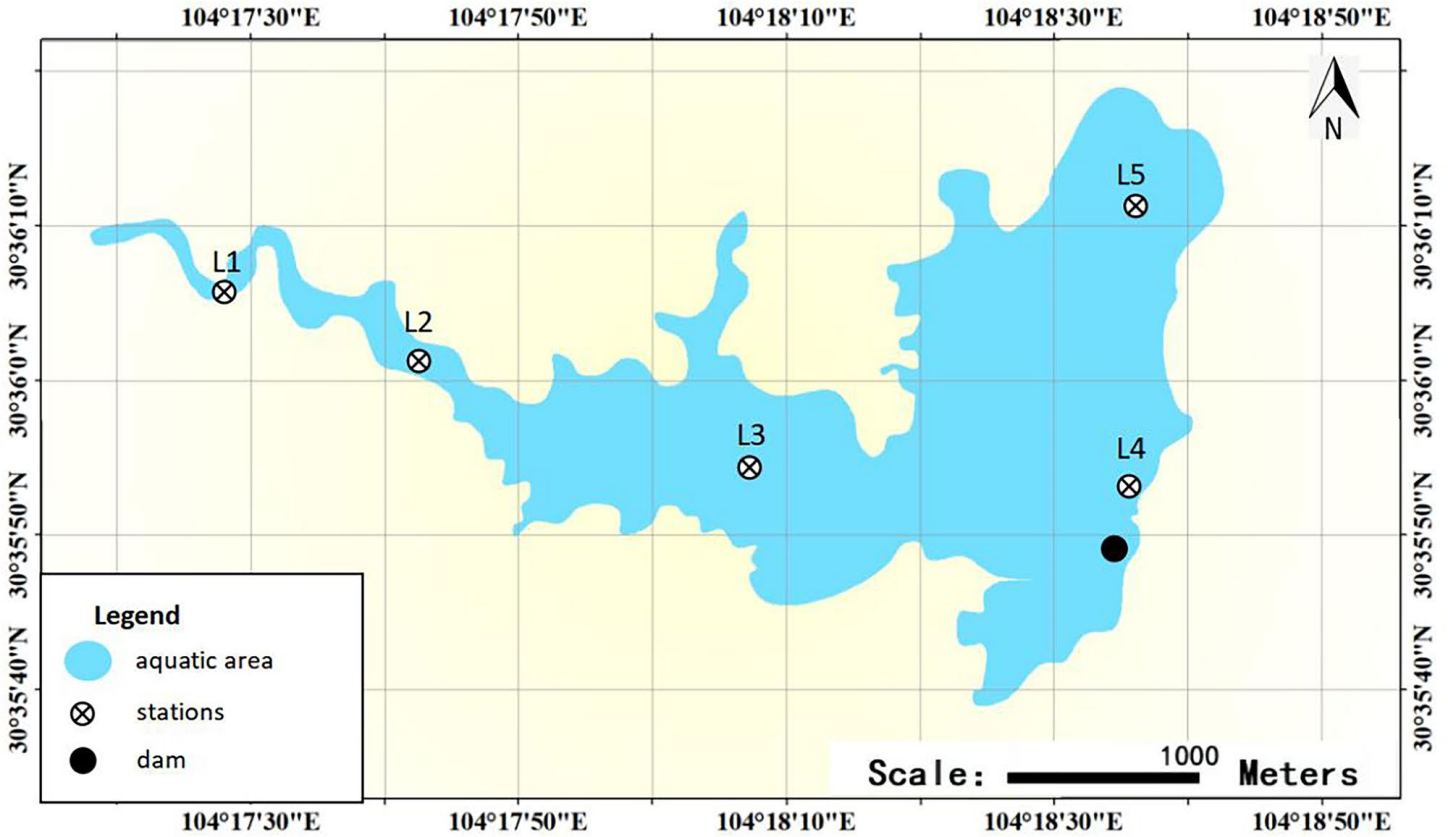
Sampling sites at Zhangjiayan Reservoir.

Based on the characteristics of the overlying water body and eutrophication status of Zhangjiayan Reservoir, a total of 5 sampling points were set up in the entire lake (Fig. 1). In spring (April), summer (July), autumn (November), and winter (January) of 2022–2023, the overlying water (5–10 cm above the sediment) was collected using an gas-tight water sampler. Each sampling point is repeated three times for sampling. The samples are then stored and transported in accordance with the relevant requirements of the “Water Quality Monitoring Specification SL219-98”. The samples containing the ice packs shall be kept in a cold storage at 4 °C for temporary storage and undergo physical and chemical analysis within 24 h. The raw water (1 L per sample) used for DNA extraction was immediately filtered through a 0.45 μm water-based polyethersulfone material microporous filter membrane to remove impurities. Subsequently, a vacuum pump was used to filter the sample through a 0.22 μm water-based polyethersulfone material microporous filter membrane. The filtered membranes were then carefully removed and stored at − 80 °C in an ultra-low temperature freezer for subsequent DNA extraction purposes.

### Measurement of water quality indicators

pH and water temperature (T) were measured on site using the HI991301 portable multi-parameter temperature measuring instrument. Dissolved oxygen (DO) was measured on site using the HQ3OD portable dissolved oxygen meter. Total nitrogen (TN), five-day biochemical oxygen demand (BOD_5_), permanganate index (COD_Mn_), and chlorophyll a (Chla) were measured according to the experimental protocol of Guo et al.^6^. Total phosphorus (TP), dissolved total phosphorus (DTP), and soluble reactive phosphorus (SRP) were measured according to the experimental protocol of Li et al.^13^.

### DNA extraction and PCR amplification

After cutting the sterile membrane used for DNA extraction into pieces with sterile scissors, the total genomic DNA was extracted using the DNA extraction kit (FastDNA Spin Kit, MP Biomedicals, Santa Ana, CA, USA) and the operating procedures were followed as instructed by the kit manual. The DNA extracted was checked by 1% agarose gel electrophoresis, and the concentration of DNA was determined using a micro-spectrophotometer (NanoDrop ND-2000, ThermoScientific, Waltham, MA, USA). The extracted DNA was stored in a freezer at − 80 °C for future use.

PCR amplification and high-throughput sequencing were performed using bacterial primers 338F: 5′-ACTCCTACGGGAGGCAGCA-3′ and 806R: 5′-GGACTACHVGGGTWTCTAAT-3′^15^. The PCR protocol was as follows: 30 cycles of initial denaturation at 95 °C for 30 s, denaturation at 98 °C for 15 s, annealing at 55 °C for 30 s, extension at 72 °C for 30 s, and then extension at 72 °C for 10 min. The PCR amplification products were subjected to agarose gel electrophoresis using a 2% agarose gel. The desired fragments were then recovered using the AxyPrep DNA Gel Extraction Kit (Axygen Biosciences, Union City, CA, USA). Each sample was repeated three times. After fluorescence quantification of the PCR products, the sequencing was performed on an Illumina’s Miseq PE300 platform (provided by Shanghai Majorbio Bio-Pharm Technology Co. Ltd., Shanghai, China).

### Data processing

The DNA was sequenced using the Illumina Miseq PE300 platform (Shanghai Majorbio Bio-pharm Technology Co., Ltd.) with paired-end reads of 300 base pairs. The original paired-end sequencing reads were subjected to quality control and assembly using the fastp software (https://github.com/OpenGene/fastp)^16^.The Dada2 algorithm in the QIIME2^17^ pipeline was employed to denoise the sequences, resulting in high-quality sequences. The sequences that have undergone Dada2 denoising are typically referred to as Amplified Sequence Variants (ASVs). In order to compare the changes in microbial communities among different varieties, all bacterial samples were subsampled at a certain sequencing depth, and subsequent analysis was performed on the subsampled bacterial samples.

### Data analysis

Statistical analysis was performed using the statistical product and service solutions (SPSS) statistical software (version 20.0, IBM, Armonk, NY, USA). Based on the Silva 16S rRNA gene database (v 138, http://greengenes.secondgenome.com), the ASVs were taxonomically classified using the Blast classifier in Qiime2 for species-level analysis^18^. The Alpha diversity indices Chao and Shannon were calculated using the software mothur^19^ (http://www.mothur.org/wiki/Calculators), and the Wilcoxon rank-sum test was performed to analyze the inter-group differences in Alpha diversity. The similarity of microbial community structure among samples was examined through NMDS analysis using the Bray–Curtis distance algorithm. The Wilcoxon multiple test was performed to conduct hypothesis testing of the species in the microbiota among multiple sample groups, in order to determine the bacterial taxa with significantly different abundance between different groups. Redundancy analysis (RDA) is used to investigate the impact of environmental factors on bacterial community structure^20^. The co-occurrence network at the genus level was visualized using Gephi software. In order to reduce the complexity of the network, only genera with a relative abundance that accounted for the top 50% and appeared in at least 50% of the samples were included. The Spearman's correlation coefficient r ≥ 0.6 was used to determine significance, with a significance level set at *P* < 0.05^21^. The heatmap analysis of the correlation between the microbial community and environmental factors was generated using Origin software (version 2021b, OriginLab, Northampton, MA, USA). Regression analysis using SPSS software (version 20.0, IBM, Armonk, NY, USA) was employed to examine the correlation between environmental factors and microbial community.

## Results and analysis

### Physicochemical properties of the overlying water

The physicochemical indicators of Zhangjiayan Reservoir are shown in Table 1. The pH value of the overlying water body ranges from 7.39 to 9.02, with the highest values observed in spring, followed by summer, and the lowest values in autumn and winter. Water temperature ranges from 9.76 to 29.09 °C, with the highest values in summer and lower values in spring and autumn, and the lowest values in winter. The range of DO is 5.09–10.55 mg L^−1^, with the highest values in summer, lower values in spring and autumn, and the lowest values in winter. The range of TN is 0.51–1.03 mg L^−1^, with higher values in summer than in spring, and lower values in autumn and winter. The range of biochemical BOD_5_ is 0.91–3.23 mg L^−1^, with the highest values in summer, followed by spring, and the lowest values in autumn and winter. The range of COD_Mn_ is 2.3–5.8 mg L^−1^, with the highest values in summer, followed by spring, and the lowest values in autumn and winter. The range of TP is 0.032–0.092 mg L^−1^, with the highest values in summer, lower values in spring and autumn, and the lowest values in winter. The range of chlorophyll a is 7.0–26.0 mg m^−3^, with the highest values in summer, followed by spring, and the lowest values in autumn and winter.

**Table 1:** Physicochemical factors of overlying water in different seasons.

Season	Spring	Summer	Autumn	Winter
pH	8.60 ± 0.12a	8.43 ± 0.59a	8.08 ± 0.57b	8.02 ± 0.63b
T (°C)	17.70 ± 5.39b	26.86 ± 2.23a	17.32 ± 1.03b	10.22 ± 0.46c
DO (mg L^−1^)	6.99 ± 0.35b	9.76 ± 0.81a	6.86 ± 0.76b	5.17 ± 0.52b
TN (mg L^−1^)	0.89 ± 0.08a	0.93 ± 0.09a	0.59 ± 0.07b	0.62 ± 0.07b
BOD_5_ (mg L^−1^)	1.38 ± 0.34b	2.28 ± 0.95a	1.10 ± 0.18b	1.08 ± 0.17b
COD (mg L^−1^)	4.5.0 ± 0.65b	5.50 ± 0.23a	4.5.0 ± 1.50b	3.40 ± 0.67c
TP (mg L^−1^)	0.066 ± 0.012a	0.087 ± 0.009a	0.053 ± 0.001b	0.048 ± 0.016b
DTP (mg L^−1^)	0.017 ± 0.002b	0.032 ± 0.012a	0.009 ± 0.007c	0.007 ± 0.002c
SRP (mg L^−1^)	0.008 ± 0.001a	0.011 ± 0.014a	0.003 ± 0.002b	0.003 ± 0.001b
Chla (mg m^−3^)	15.5 ± 2.5b	21.5 ± 4.5a	11.5 ± 1.5b	9.5 ± 2.5b

Data are means ± stand deviation. In the same row, data with different letter such as a, b, and c indicate significant differences, while data with the same letter indicated insignificant differences at 0.05 level. Data with letters ab were insignificantly different from both data with letter a and data with letter b.

### Diversity of microbial communities

This article investigates and analyzes diversity indices including ace and Chao reflecting bacterial community richness, Shannon and Simpson community diversity reflecting community diversity, and coverage reflecting sequencing depth (Table 2). The coverage of all samples was greater than 0.9, indicating that the sequencing depth was sufficient to cover most microbial species information and the sample size was sufficient to reflect the diversity differences among different communities. The bacterial diversity in Zhangjiayan Reservoir is relatively rich, with ace indices ranging from 189.39 to 4417.65, Chao indices ranging from 88.76 to 5576.85, Shannon indices ranging from 1.98 to 7.65, and Simpson indices ranging from 0.0419 to 0.0033. The diversity indices were ranked in order of magnitude as winter > autumn > summer > spring. Statistical analysis showed that the ace, Chao, Shannon, and Simpson indices in autumn were significantly different from those in spring and summer (*p* < 0.05), but not significantly different from those in winter. The ace, Chao, Shannon, and Simpson indices in winter were significantly different from those in spring and summer (*p* < 0.05). There was no significant difference in ace, Chao, Shannon, and Simpson indices between spring and summer.

**Table 2:** Bacterial diversity index in Zhangjiayan Reservoir.

Season	Point position	No. of ASVs	Ace	Chao	Shannon	Simpson	Coverage	Phyla	Classes	Orders	Families	Genera
SP	L1	391	407.50	403.78	3.9546	0.0587	0.9980	18	37	90	135	202
	L2	285	308.56	298.89	2.8433	0.1767	0.9979	17	31	72	107	159
	L3	188	189.39	88.76	2.6663	0.1754	0.9997	17	25	55	108	123
	L4	246	253.58	252.78	3.1658	0.1251	0.9989	16	29	67	99	138
	L5	382	391.54	390.77	3.9059	0.0551	0.9986	25	45	100	145	218
SU	L1	606	641.25	632.68	4.1179	0.0479	0.9961	22	41	94	145	215
	L2	726	917.31	851.82	4.2931	0.0419	0.9899	21	44	86	133	186
	L3	356	377.89	368.00	3.3598	0.0937	0.9979	15	27	133	186	227
	L4	339	382.60	366.61	1.9812	0.4475	0.9967	17	26	61	99	160
	L5	319	322.34	321.03	3.9872	0.0465	0.9994	15	27	64	99	144
AU	L1	852	4417.65	4113.29	7.3496	0.0014	0.9462	51	150	310	445	666
	L2	419	3054.76	2901.49	7.0930	0.0020	0.9714	48	145	300	425	623
	L3	466	3722.89	3465.96	7.1132	0.0027	0.9589	43	135	278	425	655
	L4	356	2028.00	2028.00	6.7464	0.0033	1.0000	41	122	284	419	632
	L5	268	5696.61	5576.85	7.6507	0.0007	0.9235	55	156	323	458	671
WI	L1	365	4417.65	4202.81	7.2960	0.0014	0.9449	50	145	309	434	618
	L2	393	3054.76	3811.18	7.2602	0.0017	0.9518	50	155	278	422	671
	L3	248	3722.89	3801.98	7.2554	0.0018	0.9519	46	134	286	441	734
	L4	481	2028.00	4384.31	7.4593	0.0010	0.9407	53	150	318	444	656
	L5	251	4094.62	3780.40	7.3325	0.0012	0.9532	49	132	301	442	664

SP, SU, AU, WI corresponds to the spring, SU corresponds to the spring, summer, autumn, and winter. This is consistent throughout the text.

### Microbial community composition

**Figure 2:**
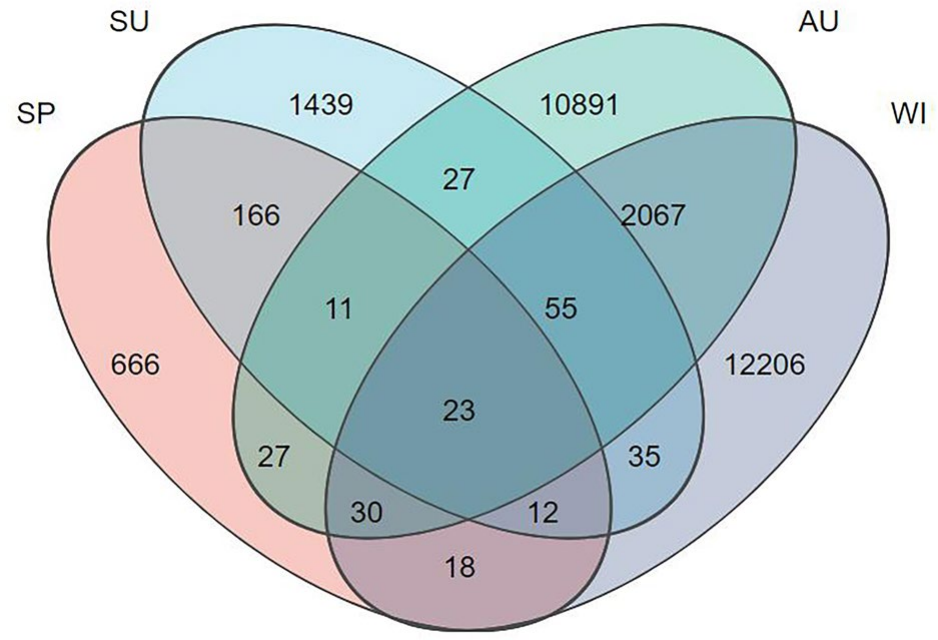
The Venn diagram of ASV of samples in spring, summer, autumn, and winter.

Four seasons, a total of 20 samples were collected at five sampling points in Zhangjiayan Reservoir. Among the 20 samples, a total of 2,142,849 valid sequences were obtained, with an average length of 416.537 bp. The range of sample sequence numbers was 31,381 to 106,886, with an average of 53,575. Among the 2,142,849 sequences, a total of 27,904 ASVs were obtained. A Venn diagram (Fig. 2) was used to compare the ASVs from the four seasons. There were 27,673 ASVs in total, with 666 unique ASVs in spring, 1439 in summer, 10,891 in autumn, and 12,206 in winter. The order of ASV abundance was spring < summer < autumn < winter.

**Figure 3:**
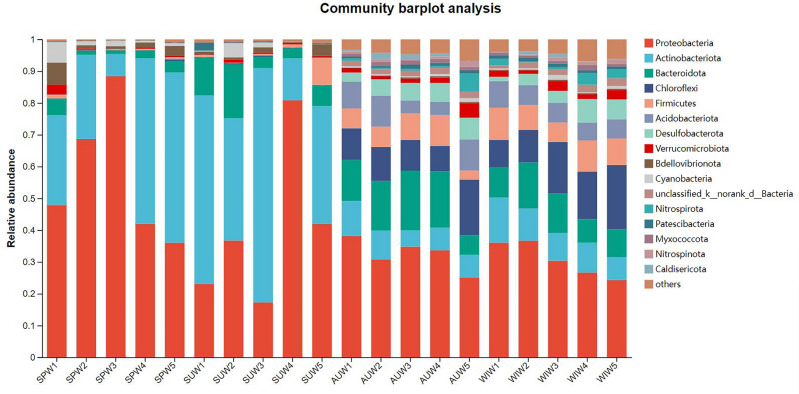
Relative abundance and composition of bacterial phyla detected in the overlying water of Zhangjiayan Reservoir, in the four seasons.

In the overlying water samples taken from the overlying water of Zhangjiayan Reservoir, a total of 66 phyla, 202 classes, 499 orders, 835 families, and 1716 genera from the bacterial domain were detected. At the phylum level, the distribution and relative abundance of the top 15 bacterial groups in the overlying water at each sampling point in Zhangjiayan Reservoir throughout the four seasons are shown in Fig. 3. The ranking of microbial populations with high abundance (average abundance > 1%) is as follows: *Proteobacteria* (relative abundance ranged from 17.26 to 88.40%, with an average abundance of 40.53%, the same below), *Actinobacteriota* (5.19–73.68%, 23.91%), *Bacteroidota* (2.26–18.41%, 9.06%), *Chloroflex* (0.06–18.32%, 8.13%), *Firmicutes* (0.05–10.40%, 5.52%), *Acidobacteriota* (0.03–8.23%, 3.69%), *Desulfobacterota* (0.01–6.64%, 2.15%), *Verrucomicrobiota* (0.01–4.37%, 1.30%), Cyanobacteria (0.04–8.13%, 1.14%). *Proteobacteria* and *Actinobacteria* dominate the microbial community in Zhangjiayan Reservoir. In spring and summer, *Proteobacteria* and *Actinobacteria* together accounted for more than 76.40% of each sample.

**Figure 4:**
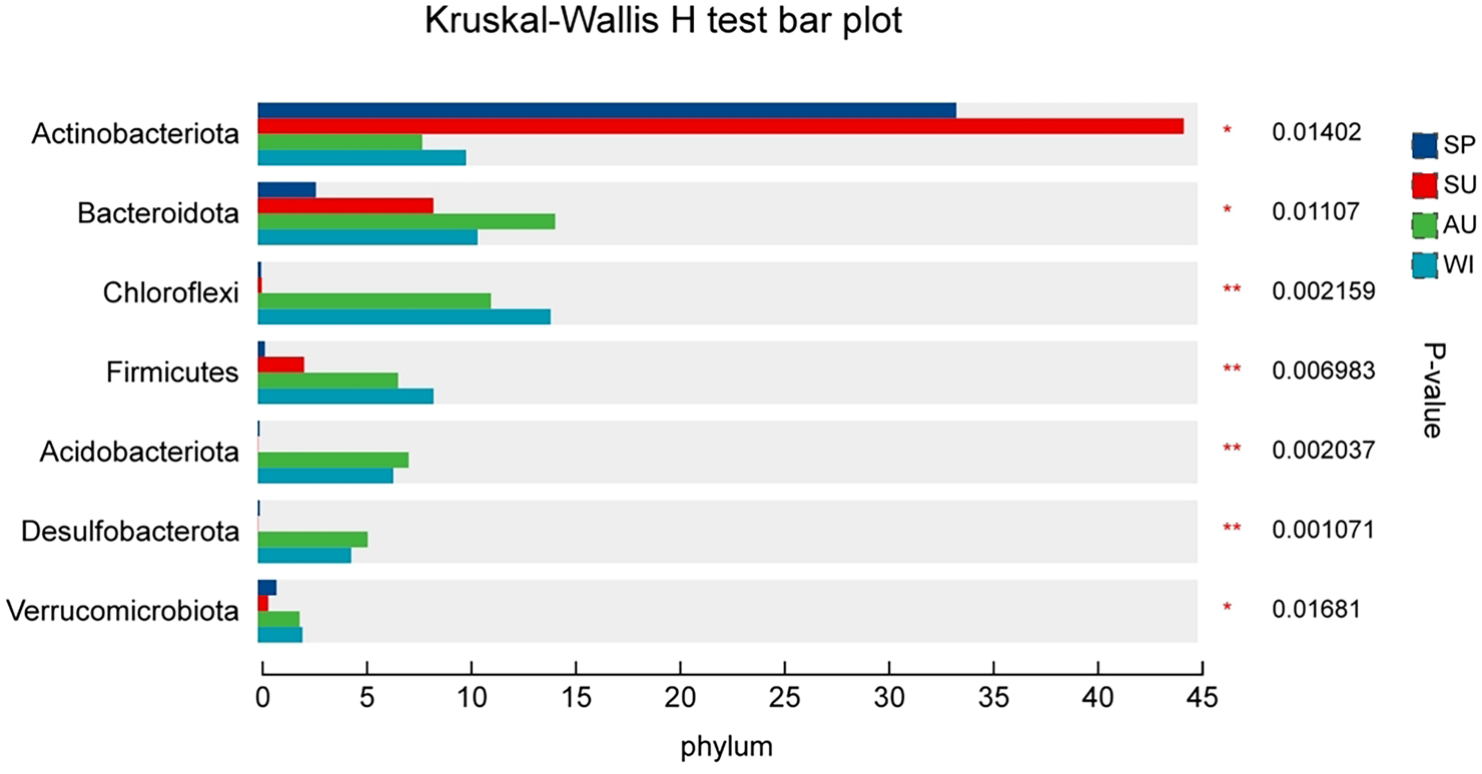
Differences in seasonal abundance distribution of bacterial taxa at the phylum level. *p < 0.05, **p < 0.01.

Abundant microbial populations were present in all four sampling seasons, but with significant differences in abundance. According to the results of the Wilcoxon multiple test (Fig. 4), there were significant differences in the mean abundance of bacterial groups with a relative abundance of > 1% among different seasons, including *Actinobacteriota, Bacteroidota, Chloroflex, Firmicutes, Acidobacteriota, Desulfobacterota,* and *Verrucomicrobiota. Actinobacteriota* had the highest abundance in summer, followed by spring, and the lowest in autumn and winter. *Bacteroidota* had the highest abundance in autumn, followed by winter, and the lowest in spring and summer. *Chloroflex* and *Firmicutes* gradually increased from spring to winter. *Acidobacteriota* and *Desulfobacterota* had the highest abundance in autumn, followed by winter, and the lowest in spring and summer. *Verrucomicrobiota* had the highest abundance in winter, followed by autumn, and the lowest in spring and summer.

**Figure 5:**
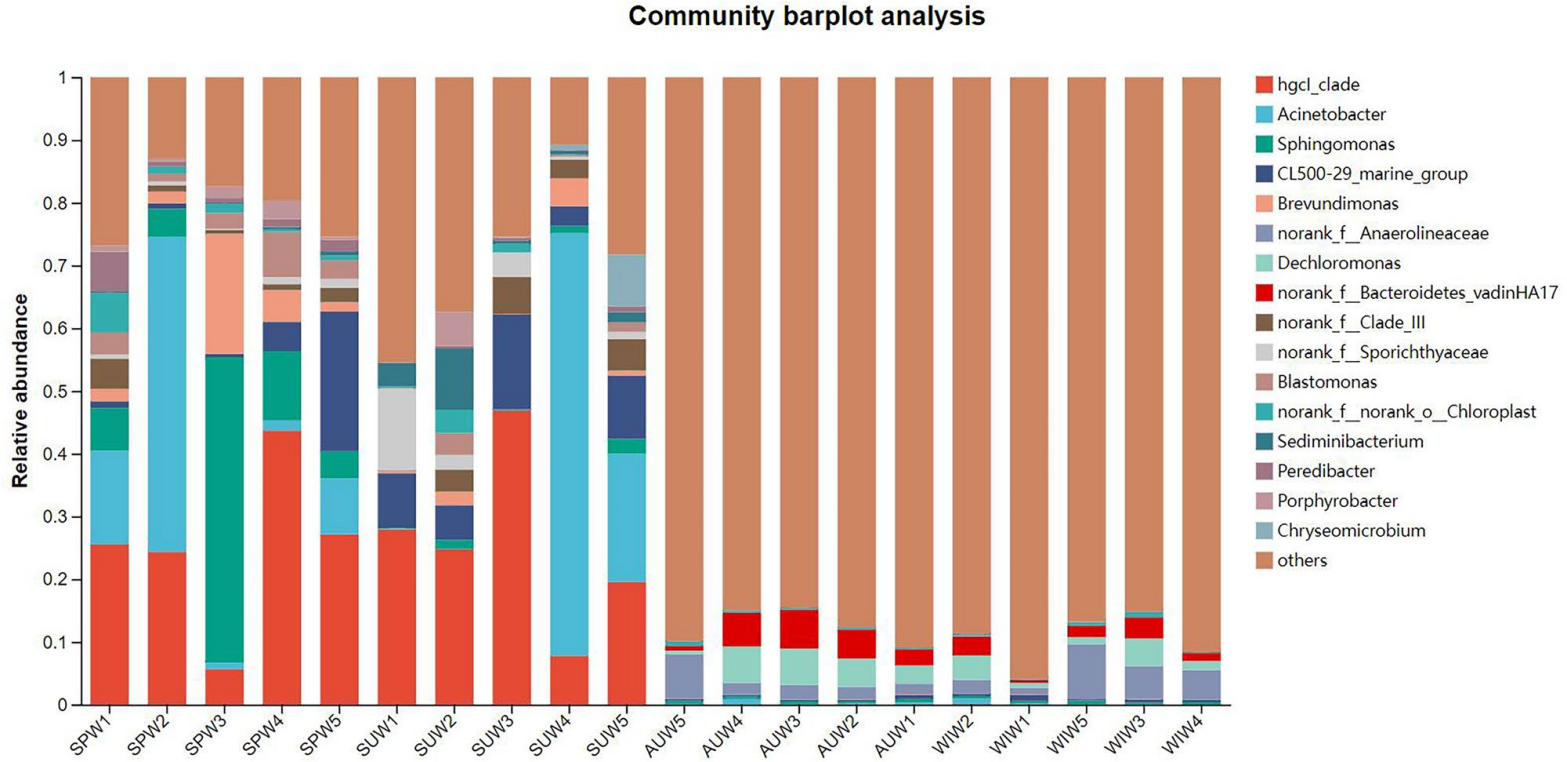
Relative abundance and composition of bacterial genus detected in the overlying water of Zhangjiayan Reservoir, in the four seasons.

At the genus level, the distribution and relative abundance of microbial populations at each sampling point in Zhangjiayan Reservoir for seasons are shown in Fig. 5. Among the 1716 genera detected in Zhangjiayan Reservoir, eight genera had a relative abundance > 1%, including *hgcI_clade* (0.12–46.53%, 19.07%), *Acinetobacter* (0.07–79.62%, 8.12%), *Sphingomonas* (0.04–49.33%, 4.78%), *CL500-29_marine_group* (0.04–22.95%, 3.27%), *Brevundimonas* (0.01–20.65%, 2.72%), ASV670 at the family level of *Anaerolineaceae* (0.01–9.95%, 1.67%), *Dechloromonas* (0.01–7.34%, 1.53%), and ASV245 at the family level of *Bacteroidetes_vadinHA17* (0.01–5.47%, 1.12%). At the genus level, there are numerous unidentified bacteria, such as ASV670 at the family level of *Anaerolineaceae* and ASV245 at the family level of *Bacteroidetes_vadinHA17*, which are present in relatively high abundance. These unidentified bacteria pose difficulties in analyzing bacterial community compositions. However, this also indicates that the diversity of microorganisms in the overlying water of Zhangjiayan Reservoir is rich, and the unknown bacterial groups contains are a valuable resource that we need to explore.

**Figure 6:**
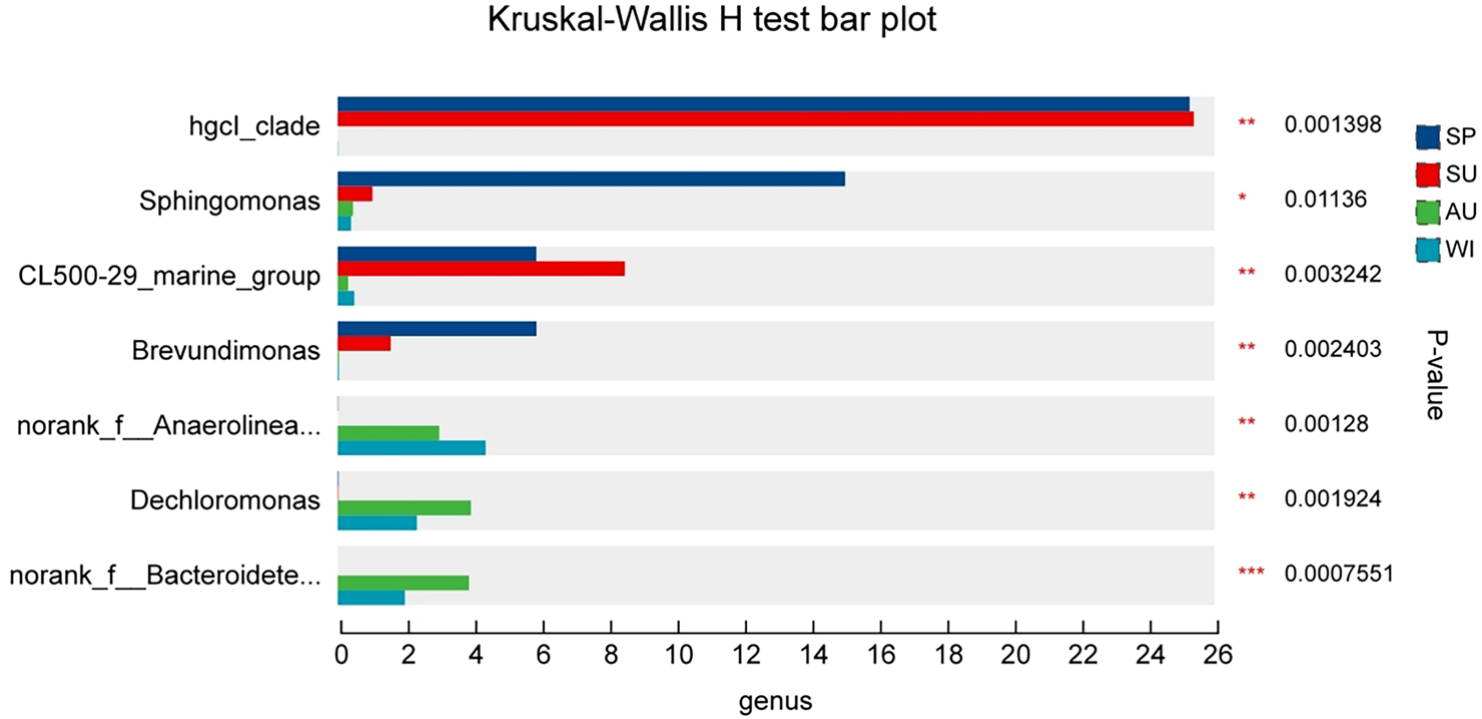
Differences in seasonal abundance distribution of bacterial taxa at the genus level. *p < 0.05, **p < 0.01.

From the results of the Wilcoxon test (Fig. 6), there were significant differences in the average abundance of S*phingomonas, CL500-29_marine_group, Dechloromonas, Brevundimonas, Blastomonas, Sediminibacterium*, and *Polynucleobacter* among the four seasons (*p* < 0.05). The *hgcI_clade* had the highest average abundance in the summer, followed by spring, and the lowest in autumn and winter. *Sphingomonas* and *Brevundimonas* gradually decreased from spring to winter. *CL500-29_marine_group* had the highest average abundance in the summer, followed by spring, and was less abundant in autumn and winter. *Dechloromonas* had the highest average abundance in autumn, followed by winter, and was less abundant in spring and summer.

### Differences in microbial community structure

**Figure 7:**
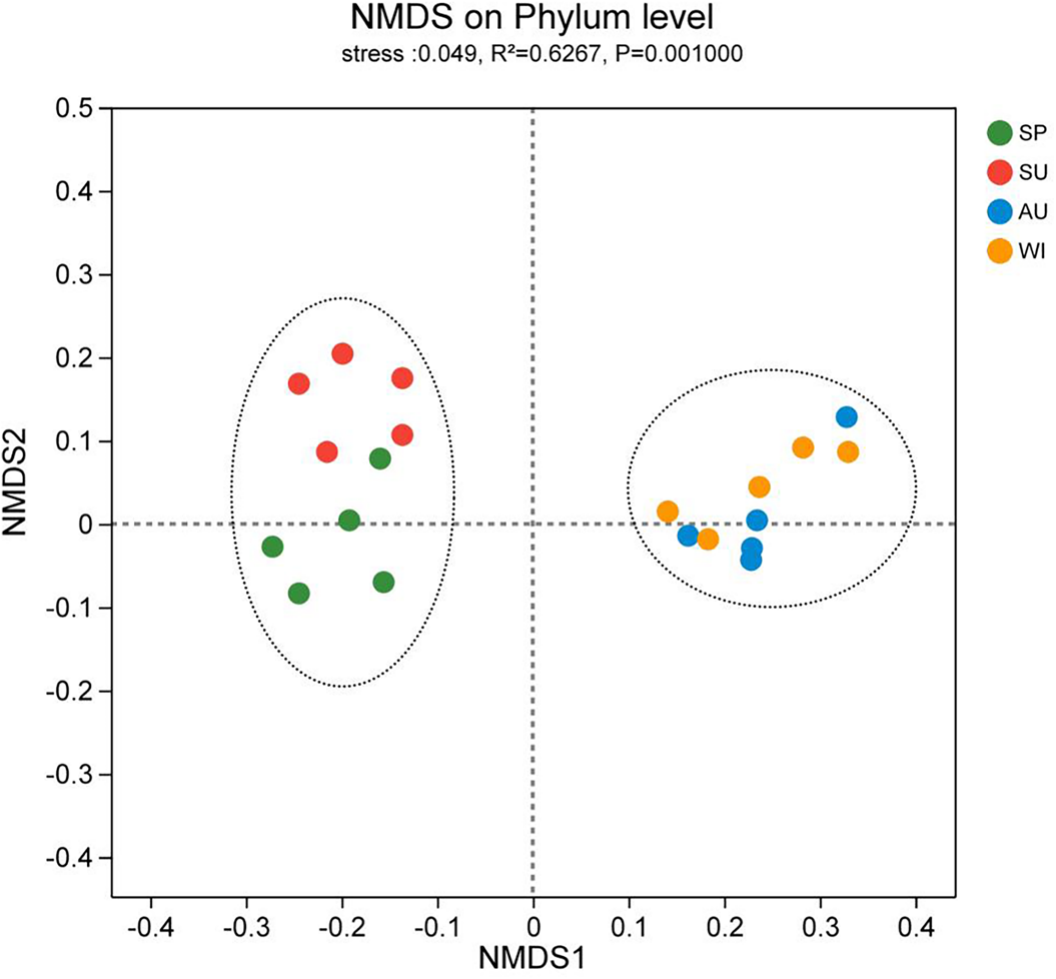
NMDS analysis of bacteria phyla and physico-chemical factors in Zhangjiayan Reservoir.

The results of evaluating the similarities and differences in microbial community composition across different samples in Zhangjiayan Reservoir based on NMDS analysis using Bray–Curtis are shown in Fig. 7. The figure reveals that the microbial communities in the spring and summer sampling points were mostly concentrated in the second and third quadrants, while those in the autumn and winter sampling points were distributed in the first and fourth quadrants, indicating a significant seasonal difference in microbial community structure. Furthermore, the microbial community structures of different samples showed high similarity in terms of spatial distribution, indicating that the impact of sampling point types on microbial community structure is limited.

**Figure 8:**
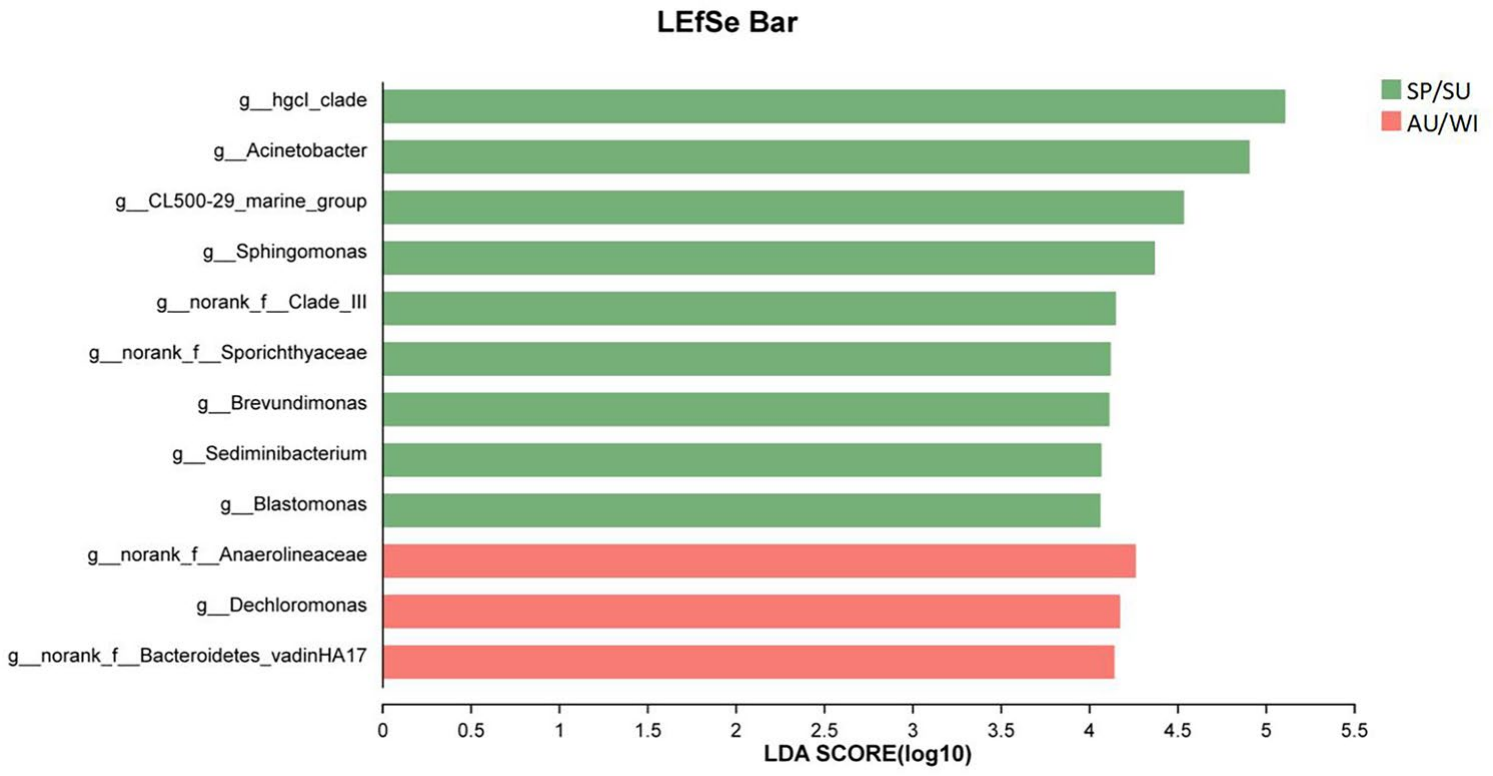
The major genera that contribute to differences in community structure.

To further identify differing bacterial species between the spring/summer and fall/winter seasons, the LEfSe method was used to calculate the differential bacterial genera in each season, as shown in Fig. 8. The primary microbial community structures that were significantly impacted by differences between the spring/summer and fall/winter seasons included *hgcI_clade, Acinetobacter, CL500-29_marine_group, Sphingomonas, CL500-29_marine_group, Brevundimonas, Sediminibacterium, Blastomonas, Dechloromonas,* as well as ASV1467 at the family level of *Clade_III*, ASV670 at the family level of *Anaerolineaceae*, ASV367 at the family level of *Sporichthyaceae*, and ASV245 at the family level of *Bacteroidetes_vadinHA17*.

### Correlation of microbial communities and environmental factors

**Figure 9:**
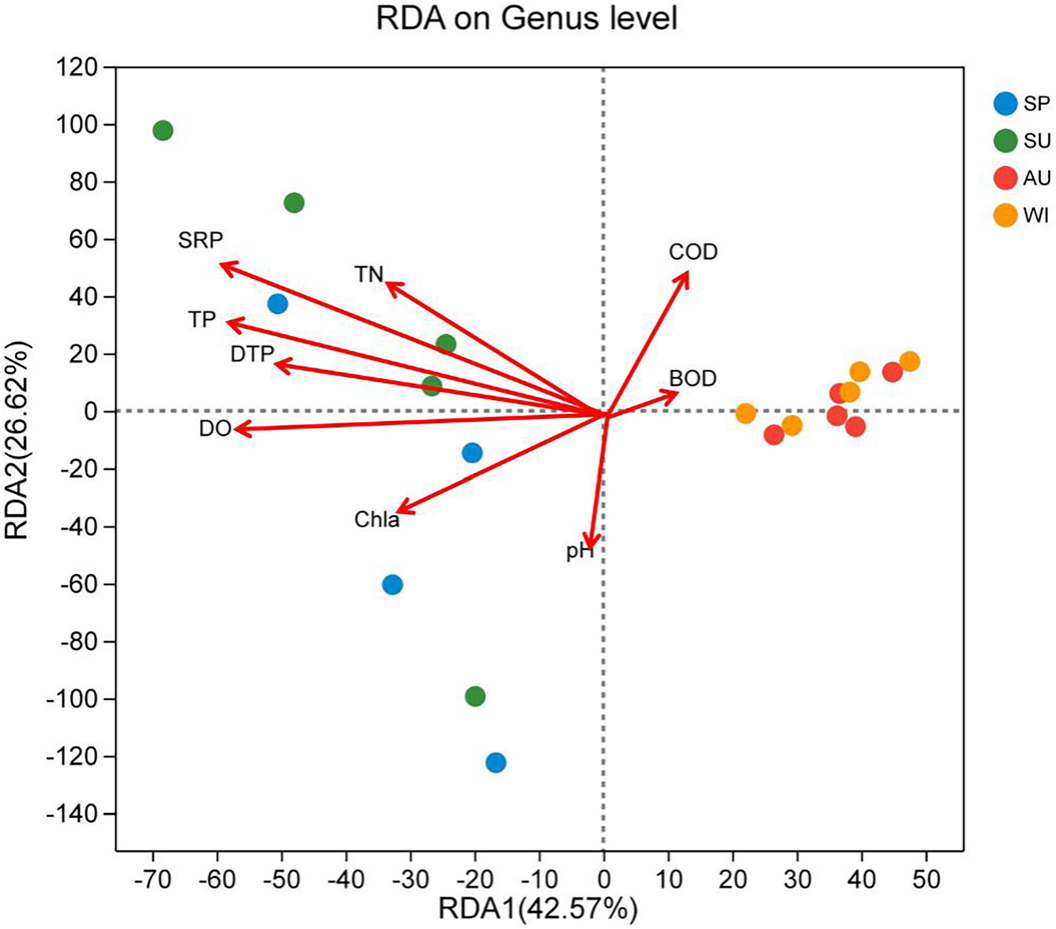
RDA analysis of bacterial genus and physicochemical factors in Zhangjiayan Reservoir.

Environmental factors were selected using the variance inflation factor (VIF) method and analyzed at the genus level using RDA. The results are shown in Fig. 9. RDA1 and RDA2 explained 42.57% and 26.62% of the community variation, respectively. Among them, TP, DTP, SRP, and dissolved DO were significantly influential environmental factors (*P* < 0.01) that affected bacterial community composition, with explanatory percentages of 36.54%, 19.28%, 55.85%, and 7.25%, respectively.

**Figure 10:**
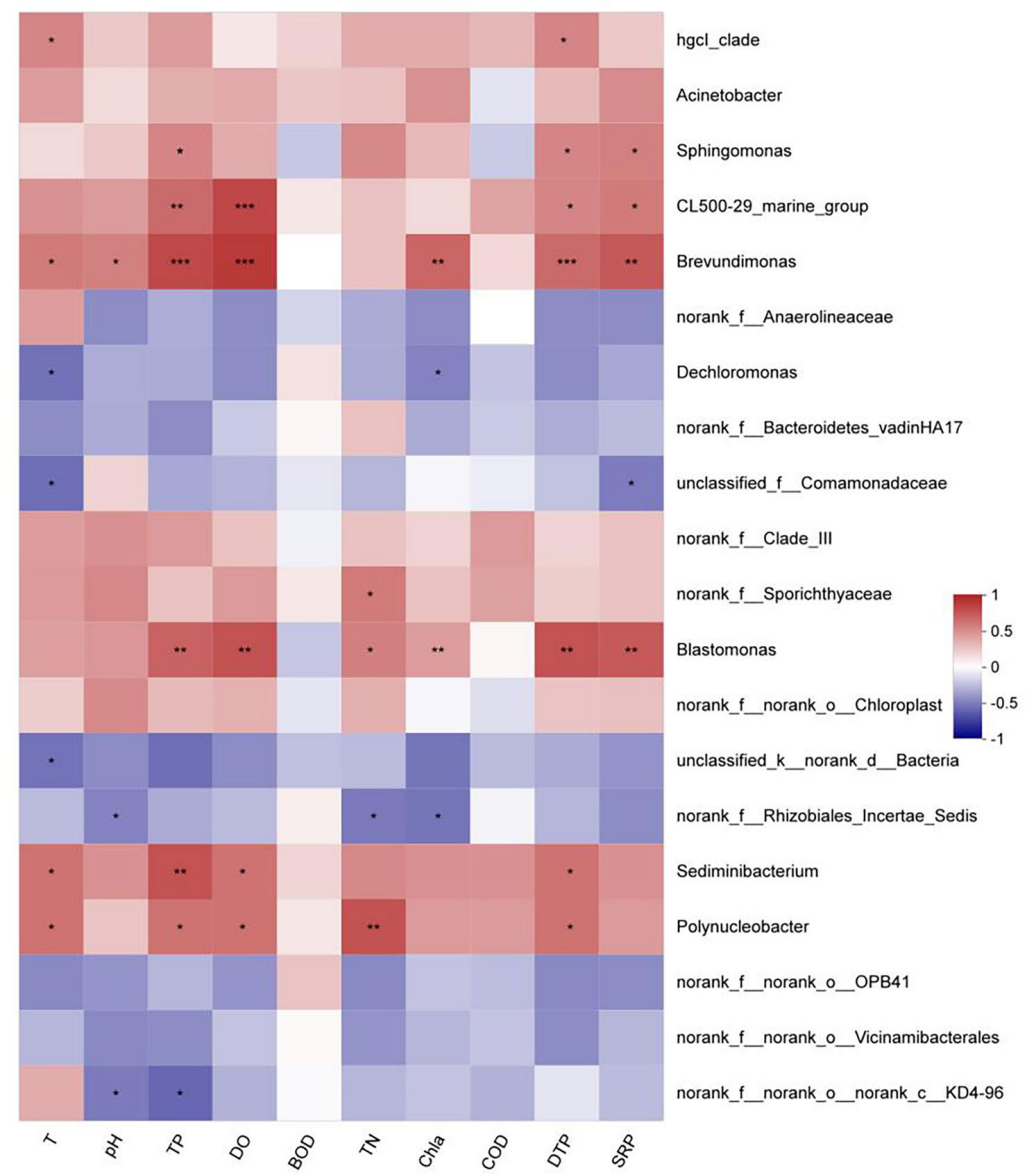
Correlation analysis of microbial populations and environmental factors at the genus level.

The correlation heatmap analysis of water physicochemical properties and the relative abundance ranking of the top 20 bacterial genera (Fig. 10) shows that temperature, pH, DO, TP, DTP, TN, Chla, SRP, and DO are significantly correlated with most dominant bacterial genera (*P* < 0.05). Among them, temperature was significantly positively correlated with *hgcl_clade*, *Brevundimonas, Sediminibacterium,* and *Polynucleobacter* (*P* < 0.05), and significantly negatively correlated with *Dechloromonas*, ASV764 at the family level of *Comamonadaceae*, and Bacillus (*P* < 0.05). pH was significantly positively correlated with *Brevundimonas* (*P* < 0.05), and significantly negatively correlated with *norank_f_Rhizobiales_Incertae_Sedis* and *norank_f_norank_o_norank_c_KD4-96* (*P* < 0.05). TP was extremely positively correlated with *CL500-29_marine_group*, *Brevundimonas, Blastomonas,* and *Sediminibacterium* (*P* < 0.01), significantly positively correlated with *Sphingomonas and Polynucleobacter* (*P* < 0.05), and significantly negatively correlated with *norank_f_norank_o_norank_c_KD4-96* (*P* < 0.05). DO was extremely positively correlated with *CL500-29_marine_group, Brevundimonas, and Blastomonas* (*P* < 0.01), and significantly positively correlated with *Sediminibacterium* and *Polynucleobacter* (*P* < 0.05). TN was extremely positively correlated with *Polynucleobacter* (*P* < 0.01), significantly positively correlated with *norank_f_Sporichthyaceae* and *Blastomona*s (*P* < 0.05), and significantly negatively correlated with *norank_f_Rhizobiales_Incertae_Sedis* (*P* < 0.05). Chla was extremely positively correlated with *Brevundimonas* and *Blastomonas* (*P* < 0.01), and significantly negatively correlated with *Dechloromonas* and *norank_f_Rhizobiales_Incertae_Sedis* (*P* < 0.05). DTP was extremely positively correlated with *Brevundimonas* and *Blastomonas* (*P* < 0.01), and significantly positively correlated with hgcl_clade, *CL500-29 marine_group, Sphingomonas, Sediminibacterium*, and *Polynucleobacte* (*P* < 0.05). SRP was extremely positively correlated with *Brevundimonas* and *Blastomonas* (*P* < 0.01), significantly positively correlated with *CL500-29 marine_group* and *Sphingomona*s (*P* < 0.05), and significantly negatively correlated with ASV764 at the family level of *Comamonadaceae* (*P* < 0.05).

### Microbial co-occurrence network

**Figure 11:**
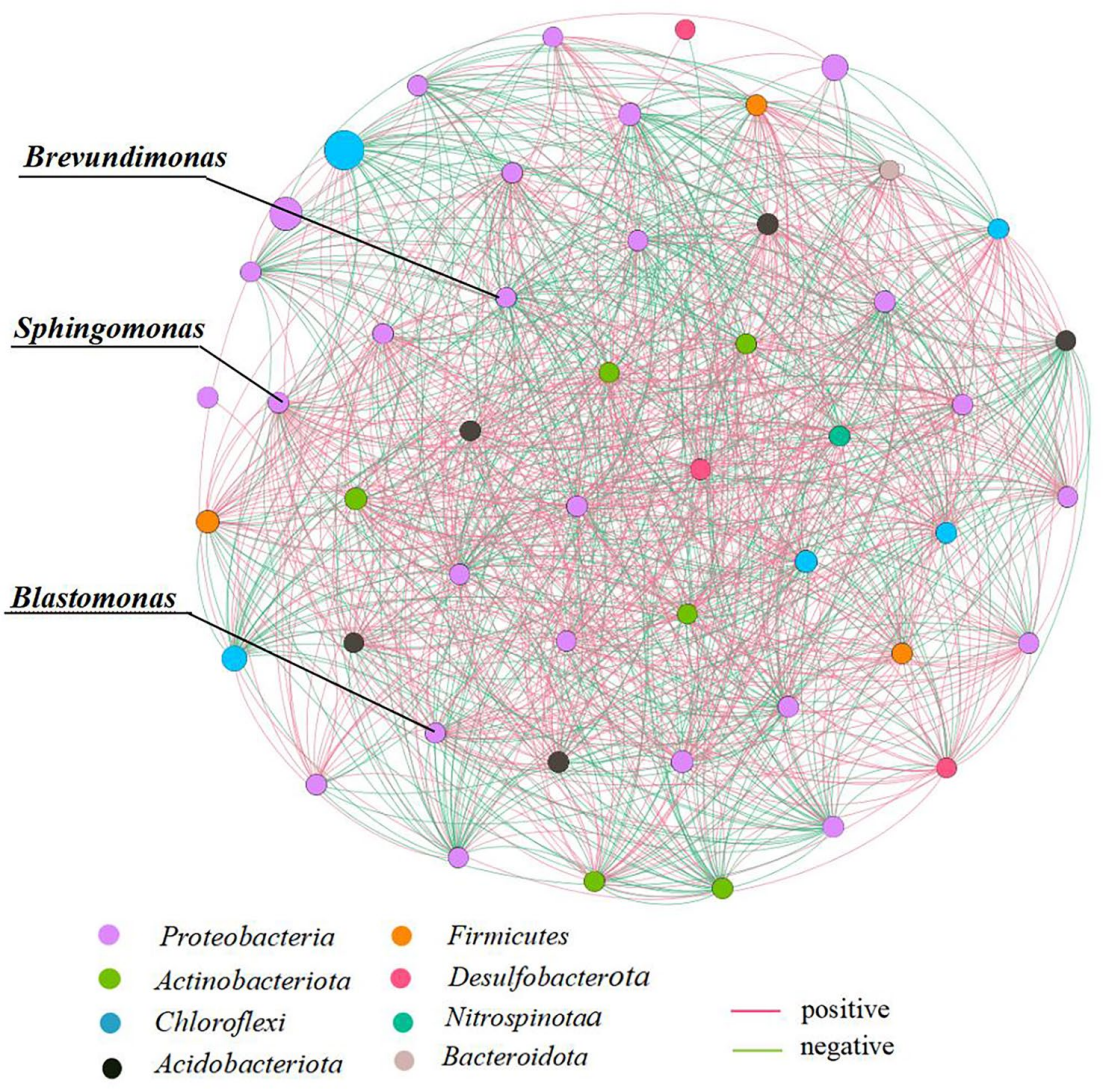
Microbial correlation network diagram Correlation between relative abundance of core bacterial groups and SRP content.The size of the nodes in the figure represents the abundance of species.

The microbial correlation network at the genus level consists of 50 nodes and 935 edges (Fig. 11). The network has an average connectivity of 38.163, an average path length of 1.228, and an average clustering coefficient of 0.932. The proportion of positive and negative correlations in the bacterial network is 52.45% and 47.55%, respectively. Based on the topological properties of the co-occurrence network, *Sphingomonas*, *Brevundimonas*, and *Blastomonas* play central roles in the network, with specific parameters shown in Table 3.

**Table 3:** Network co-occurrence topology parameters of the genus Centrolobium in the internet center.

Network indexes	*Brevundimonas*	*Blastomonas*	*Sphingomonas*
Links	32	34	35
Positive correlation	18	18	19
Negative correlation	14	16	16
Clustering coefficient	0.9112	0.9324	0.9112
Degree_Centrality	0.9167	0.8958	0.9167
Closeness_Centrality	0.9056	0.8889	0.9056
Betweenness_Centrality	0.0051	0.0039	0.0051

## Discussion

### Microbial diversity and composition

The dominant bacterial groups in the overlying water of Zhangjiayan Reservoir were *Proteobacteria* and *Actinobacteria*. Other scholars have studied the microbial community structure of lakes and reservoirs such as Lake Mar^22^, and Beihai Lake^9^, and also found that *Proteobacteria* and *Actinobacteria* were the main phylum in the water samples. The composition of dominant bacterial groups in lakes at different spatial scales was similar. *Proteobacteria*, *Actinobacteria*, or the main bacterial community in lake waters. At the dominant genus level, *hgcI_clade, CL500-29_marine_group* of the phylum *Actinobacteria*, are likewise frequently found to be the dominant population in river, lake, and reservoir water^23,24^. *Acinetobacter* and *Sphingomonas* of the phylum *Proteobacteria* are also often reported to occur in lake-reservoir water ecosystems. *Acinetobacter* is widely distributed in water bodies and soils, and it has been suggested that it is a common phosphorus-related genus in ecosystems^25^. *Sphingomonas* have the ability to metabolize a variety of carbon sources and are common bacteria that have a certain conversion effect on nitrogen^26^.

The water overlying body on the Zhangjiayan Reservoir has a rich microbial diversity. A total of 66 phyla, 202 classes, and 1716 genera in the bacterial domain were detected in the water body on the Zhangjiayan Reservoir. Compared with other lakes or reservoirs (such as Lake Taihu^27^and the Chetelson Reservoir^28^), the water body on the Zhangjiayan Reservoir exhibits a higher diversity.

### Seasonal changes in microorganisms and their response to environmental factors

Microbial communities in the water overlying body of Zhangjiayan Reservoir exhibit different levels of bacterial diversity between the spring/summer and autumn/winter seasons. Guo et al.^29^ also found a lower microbial diversity index in the spring/summer seasons compared to the autumn/winter seasons in a study of microbial communities in a drinking water source in Shanghai. In contrast to the results of this study, Zhu et al.^30^ found that bacterial diversity was highest in the spring and lowest in the autumn when studying bacterial community structures in Lake Taihu. Sun et al.^31^ found that the abundance and diversity of bacteria in the water body of Guanting Reservoir were higher in the summer, while both abundance and diversity decreased in the autumn. The diversity index of microorganisms in the overlying water body of Zhang Jiayan Reservoir in spring and summer is lower than that in autumn and winter, which may be due to the fact that spring and summer are in the rainy season and there is a period of agricultural irrigation drainage, resulting in strong water flow and uniform water quality at each sampling point, with similar bacterial community types between sampling points. In contrast, during the autumn and winter seasons, the poor water circulation capacity and uneven distribution of nutrients such as phosphorus throughout the entire lake result in higher bacterial community diversity between sampling points.

This study found that the microbial community structure in Zhangjiayan Reservoir exhibited significant seasonal variations throughout the four seasons, especially with notable differences (*p* < 0.05) between the microbial community structures during the spring/summer seasons compared to those in the autumn/winter seasons. Similarly, Pascaline Nyirabuhoro et al.^32^ analyzed the planktonic bacteria in the surface water of Xinglin Bay reservoir, a subtropical urban area in southeastern China. Through seasonal sampling, the study identified different seasonal succession patterns of planktonic bacterial communities in lakes and reservoirs, with significant seasonal variations. Furthermore, Nyirabuhoro et al.^33^ also studied the dynamic of microbial communities in East Town, Tingxi and Shidou Reservoirs in southeastern Fujian Province, China, and found four different bacterial community successions that corresponded well with four different seasons. These works indicate that different environmental variables shape microbial communities at different continuous time scales.

Correlation analysis between microbial communities and environmental factors showed that TP, DTP, SRP, and dissolved oxygen are the main factors influencing the seasonal variations of microbial community structure in water bodies. Niu et al.^11^ found that TN, TP, and transparency were the main factors affecting the microbial community in Dongting Lake. Huang et al.^8^ discovered that the microbial community in Poyang Lake was greatly influenced by TN and TP. Similar conclusions were drawn about the lakes like Dongting Lake and Poyang Lake, where nutrient concentrations mainly affect the microbial community. The eutrophication of water bodies caused by environmental factors such as phosphorus has shaped the microbial community structure in the overlying water of Zhangjiayan Reservoir.

Seasonal variations in the microbial structure composition of the overlying water body at Zhangjiayan are significantly influenced by environmental factors such as phosphorus. The results of the study indicated that the main genera responsible for differences in microbial community structures between spring and summer, and autumn and winter, include *hgcI_clade, Acinetobacter, CL500-29_marine_group, Sphingomonas, CL500-29_marine_group, Brevundimonas, Sediminibacterium, Blastomonas* during spring and summer, as well as ASV1467 at the family level of *Clade_III* and ASV367 at the family level of *Sporichthyaceae*. These dominant groups are mainly positively correlated with environmental factors, indicating that an increase in nutrient concentration in the water is more conducive to the growth and reproduction of dominant species in the bacterial community. The possible reason is that these dominant groups, such as *Hgcl_clade* and *CL500-29_marine_group*, have a close relationship with plankton, especially *cyanobacteria*, and are more adapted to grow and reproduce in high-nutrient water bodies^34^.

### Role of keystone species in microbial communities and eutrophication

Results generated by the network indicate that positive links dominate the interactions among bacterial groups in the overlying water of Zhangjiayan Reservoir. This demonstrates the importance of cooperative interactions among microorganisms in the habitat of Zhangjiayan Reservoir, with most bacteria resisting external environmental disturbances through cooperative relationships with other species. Similarly, He et al.^35^ investigated the bacteria and archaea in the South China Sea, showing that the most significant correlation in the DNA-based bacterial and archaeal networks are positive, with proportions of 77.5% and 82.61% respectively. The dominant positive connections in the networks are likely to be explained by cooperation between species, indicating that the survival mode of the microbial community in Zhangjiayan Reservoir is the result of long-term co-evolution and mutualistic symbiosis^36^.

*Brevundimonas*, *Blastomonas*, and *Sphingomonas* as central species in the microbial co-occurrence network of Zhangjiayan Reservoir, have a significant impact on interspecies interactions and community stability. Interactions between microbial communities play a crucial role in maintaining ecosystem function and structural stability. Interactions among bacterial community species to some extent affect the composition of bacterial communities and play an important role in maintaining ecosystem function and structural stability^37^. As central species, *Sphingomonas*, *Brevundimonas*, and *Blastomonas* were also predominant species with relative abundances in the top 20 in the overlying water of Zhangjiayan. We speculate that they play important roles in the seasonal variation of interspecies interactions and community structure.

**Figure 12:**
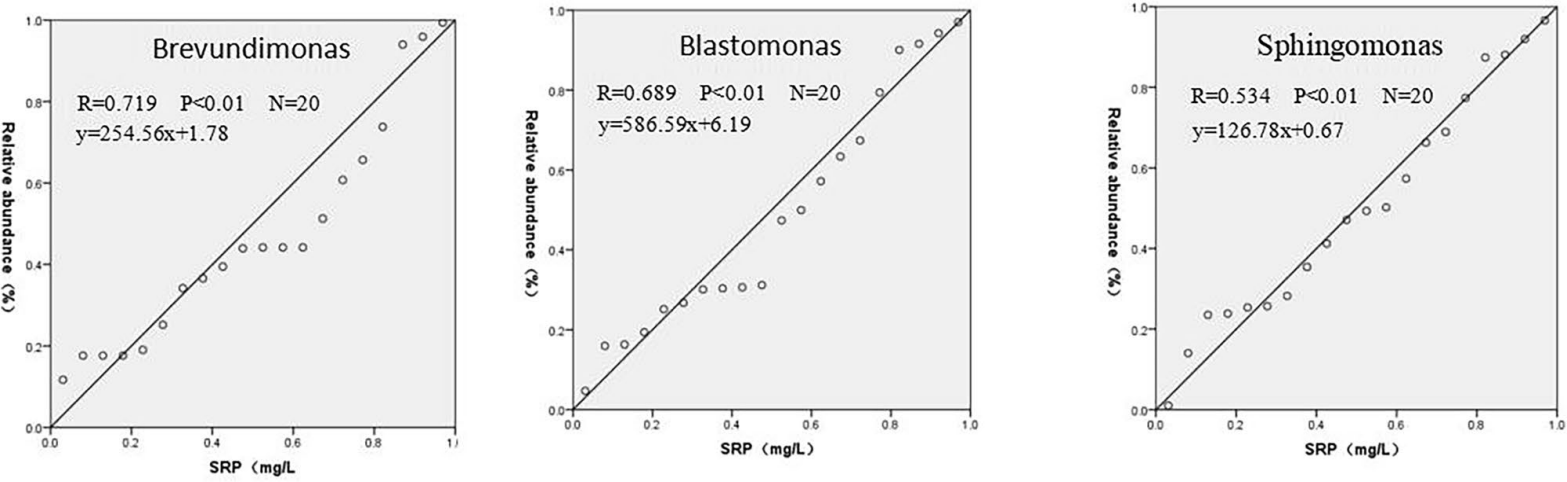
Correlation between relative abundance of core bacterial groups and SRP content.

*Brevundimonas*, *Blastomonas*, and *Sphingomonas* are central species in the microbial co-occurrence network of Zhangjiayan Reservoir, where they play important roles not only in community stability and interspecific interactions, but also in material cycling processes, especially in the phosphorus cycle. Bacteria are important mineralizers of organic phosphorus, as they can mineralize organic phosphorus into orthophosphate, participating in the chemical cycling of phosphorus and maintaining the eutrophication status of lakes^38^. *Brevundimonas* is a common genus of phosphate-related bacteria in ecosystems ^39^. Although *Sphingomonas* and *Blastomonas* have few reports related to phosphorus, we speculate that they may play an important role in the transformation of phosphorus forms. Phosphorus is typically the first limiting nutrient for primary productivity in lakes, with the phosphorus available directly to plankton being SRP. Therefore, SRP is an important factor in determining the nutrient status and productivity of lakes^40^. In order to further demonstrate the important impact of *Brevundimonas*, *Blastomonas*, and *Sphingomonas* on eutrophication of the Zhangjiayan Reservoir water body, linear regression analysis was conducted between these bacteria and SRP in overlying water. The results showed that the relative abundance of *Brevundimonas*, *Blastomonas*, and *Sphingomonas* was significantly positively correlated with SRP content (*p* < 0.01). This indicates that these bacteria may play an important role in the transfer and transformation of phosphorus at the sediment–water interface. Phosphorus in the sediment is decomposed or mineralized by bacteria, releasing SRP into the overlying water, causing eutrophication of the lake. Therefore, these bacterial groups play an important role in the process of phosphorus migration and transformation at the sediment–water interface, which has a significant impact on eutrophication in the Zhangjiayan Reservoir. Tong et al.^41^ also similarly found that the microbial communities in Lake Chaohu are conducive to maintaining or further accelerating the eutrophication process in lakes. Therefore, we speculate that *Brevundimonas*, *Blastomonas*, and *Sphingomonas* act as the central nodes in the microbial co-occurrence network of Zhangjiayan Reservoir, significantly impacting the eutrophication of the reservoir water (Fig. 12).

## Conclusions

A total of 66 phyla, 202 classes, 499 orders, 835 families, 1716 genera, and 27,904 ASVs of the bacterial domain were detected in the 20 overlying water samples collected from the Zhangjiayan Reservoir. The microbial community composition exhibited a high level of diversity. *Actinobacteria* and *Proteobacteria* dominate the microbial community in Zhangjiayan Reservoir. At the genus level, *hgcI_clade* has the highest abundance, making it the dominant population. The bacterial community structure in spring/summer and autumn/winter shows significant differences under the influence of seasonal environmental factors. Total phosphorus, dissolved total phosphorus, soluble reactive phosphorus, and dissolved oxygen are the main environmental factors that affect the microbial community structure of Zhangjiayan Reservoir. *Sphingomonas, Brevundimonas*, and *Blastomonas*, as central species in the overlying water of Zhangjiayan Reservoir, play important roles not only in community stability and interspecies interactions, but also in material cycling processes, especially in the phosphorus cycle. They may have a significant impact on the eutrophication of Zhangjiayan Reservoir water.

## Data Availability

The datasets analysed during the current study are available in the NCBI repository (https://www.ncbi.nlm.nih.gov/). The accession is PRJNA1045237.
